# Patterns of Living Lost? Measuring Community Participation and Other Influences on the Health of Older Migrants in China

**DOI:** 10.3390/ijerph19084542

**Published:** 2022-04-09

**Authors:** Xiangjing Zhang, Wusi Zhou, Biya Jiang

**Affiliations:** 1 School of Public Administration, Hangzhou Normal University, Hangzhou 311121, China; 20030117@hznu.edu.cn (X.Z.); jiangbiya@stu.hznu.edu.cn (B.J.); 2 Center for Population and Development Policy Studies, Fudan University, Shanghai 200433, China

**Keywords:** community activities, care services, health status, human capital, environmental characteristics, older adults

## Abstract

Community participation is a key element of active aging that promotes a new paradigm to enhance health and well-being as people age. However, social isolation is often a concern for older migrants. In this study, we aimed to investigate the current status of older migrants’ community participation and assess the main influences on three forms of welfare, development, and organizational participation. We adopted a quantitative research design for this study. A questionnaire survey was completed by 1216 older migrants in 4 cities; 1105 valid responses were received, representing a response rate of around 91%. The research findings showed that the current participation of older migrants in community activities was limited. By comparison, full self-care capability and non-chronic illness positively affected general and welfare participation. Educated at primary school had a negative influence on general, development, and organizational participation, whereas knowledge of the local language was a significant predictor of general and development participation. Urban inclusion and resident friendship had positive effects on general, welfare, and development participation. The study also revealed direct influences of socioeconomic characteristics on different types of participation. Moving forward, actions are needed to maximize older migrants’ participation in public events and community life.

## 1. Introduction

With the aging of societies and the globalization of migration, the number of older migrants around the world is growing. In mid-2020, there were approximately 34.30 million migrants aged 65 and over, accounting for 12.2% of the total migrants worldwide [1]. In China, driven by the implementation of urbanization strategies, the number of older migrants has been increasing rapidly. The Seventh National Population Census of China 2020 revealed that the migrant population and the older population aged 60 and over have reached 376 million and 264 million, accounting for 26.62% and 18.70% of the total population, respectively. Based on the proportion of older migrants aged 60 and over in 2015, the number was estimated to reach 27 million in 2020. Following migration, older adults often face a range of problems, such as adaptation to an unfamiliar environment and cultural integration [2,3,4]. Caidi et al. reported in qualitative research that migrants at old age found difficulties in learning a new language and faced anxieties in understanding unfamiliar practices [5]. A study by Torres showed that older migrants experienced cultural conflicts and civil disruption caused by a lack of information resources and local networks [6]. Those who are unprepared to integrate into a new culture and local language with little social support are more likely to experience community isolation and health impairment [7,8,9,10]. Klok et al.’s investigation found that there was a positive correlation between belonging and loneliness; older migrants with a lower sense of belonging were more likely to be lonely [11]. Dolberg et al. explored the complex relationships between the length of migration and the level of loneliness and suggested that long-term migrants reported lower feelings of loneliness [12]. Mui and Lee highlighted that older immigrants were vulnerable to social isolation associated with higher anxiety and depression [13]. To address these issues, it is crucial to promote older migrants’ participation and resocialization.

“Participation” is recognized as one of the three pillars of active aging by the World Health Organization (WHO). It highlights older people’s right to exercise their participation in society, economy, culture, civil affairs, etc. [14]. From a sociological perspective, participation means getting involved in specific, collective, voluntary actions in a society or a community, leading to goal achievement and personal fulfillment [15]. Accordingly, community participation is conceptualized as interactions with others in typical civic, recreational, and other social activities in the community [16]. Older migrants’ inclusion in all areas of community life has been regarded as a key element of an age-friendly city [17]. Methodologically, “community” represents an overall abstract “society” and the spatial and temporal location of people’s lives [18]. Analysis of community participation can objectively uncover the extraction of resources, the network of relationships, and the identification of local citizenship among older migrants, reflecting the age group’s actual life in a new environment. Previous studies have empirically demonstrated that promoting older migrants’ participation in community activities is vitally important to achieve longer healthy life expectancy, as well as better quality of life. For example, participating in local religious activities was found to be a benefit to psychological well-being [19,20,21], and actively seeking community healthcare services and other social support can reduce feelings of alienation and isolation [3,22]. Joining social organizations and other programs; attending immigrant language classes and housing services; and obtaining “reciprocal” neighborhood support through childcare, housekeeping, and volunteering can strengthen the sense of belonging and community [2,4,23].

In China, there are two flows of migration in the context of urbanization; one is city-to-city migration, and the other is interprovincial migration. To improve the participation of these internal migrants at an older age, the Chinese government published a couple of policies to protect their right to community participation. The Law of the People’s Republic of China on the Protection of the Rights and Interests of the Elderly (2018 Amendment) highlights that older migrants should be given equal privileges. The National Basic Public Health Service Specification (2017) confirms that basic public health services should be given to all residents within a district. The 2017 State Council’s Opinions on Strengthening and Improving Community Governance in Urban and Rural Areas calls for improvement of service provision across local communities. Older adults have gradually become the main force of the whole society. Furthermore, the Notice of the Ministry of Civil Affairs on Further Strengthening the Establishment of Elderly Associations in Urban and Rural Communities (2015) and the Elderly Education Development Plan (2016–2020) specifies the rights of older migrants to participate in local associations and community-based education programs. The Opinions of the Ministry of Civil Affairs on Strongly Cultivating and Developing Community Social Organizations (2017) requires more opportunities for migrants to participate in local governance and to integrate into local community. This underlines the right of older migrants to participate in public affairs in their local communities.

Although social policies encourage older migrants’ community participation, very few studies have focused on this area. In particular, related studies mainly examined older migrants’ social integration [24,25], voluntary services [26,27,28,29,30], adaptation to a new living environment after migration and resettlement [31], and adaptation to an unfamiliar society [32]. Therefore, with this study, we aim to fill this gap by investigating internal older migrants’ participation in community activities and its key influences. We seek to address three central research questions: (i) what is the current status of older migrants’ community participation; (ii) which factors have influenced different forms of community participation among older migrants; and (iii) how to improve older migrants’ participation in the local community.

## 2. Conceptual Framework for Community Participation

### 2.1. Elements of Community Participation

There are different types of community participation, typically divided into three or four categories. The three categories include community public affairs, volunteering, and recreational and sport activities [28]; informal social connection, civic engagement, and political participation [33]; recreation and sports, social welfare, and public management [24]; and public welfare and entitlement [25]. Meanwhile, the four categories consist of mandatory, guiding, spontaneous, and planned participation [34]. In this study, community participation refers to different social activities taking place within the community domain, as highlighted by Vaughan et al. [35]. For older migrants, this means participation in community activities in the public space.

Doyal demonstrated the importance of participation and its requisites in terms of basic human needs [36]. He pointed out that successful community participation is necessary for individuals to achieve certain success, with health and autonomy as required conditions or resources. Health is a state of complete physical and mental well-being; diseases and disabilities can contribute to limitations to participating in community activities. Autonomy means spontaneous activity that puts ideas and actions into practices. It involves three requirements: the first is “understanding”, with the capability of social interaction and verbal communication; the second is mental health, with the ability to understand emotional and cognitive knowledge; the third is opportunity, which allows people to participate in certain lifestyles. It is natural for a person to participate in different activities when they have the physical, intellectual, and emotional capacity to interact with others, whereas a lack of health and autonomy might lead to a failure of social participation. It is also crucial to have an open and inclusive environment in migrant communities, which can provide opportunities for participation and interaction. To be specific, each migrant community can recognize the importance of equality and diversity, and there are relevant policies in place to offer a holistic response to the multi-disciplinary needs of the older migrant population [14,35]. Meanwhile, new relationships with the general local population play an important role in the health and well-being of older migrants, as they can be a source of support or stress [7,33].

Therefore, to promote social participation, three elements are necessary. Firstly, health serves as the basis for social participation, indicating physical, emotional, and cognitive well-being. Secondly, ability is a prerequisite, meaning the ability and skill to engage in participation with certain human capital. Thirdly, opportunity is a condition, referring to an inclusive living environment, which can provide opportunities for participation. As a result, in this study, we analyzed older migrants’ community participation from three perspectives: health status, human capital, and environmental characteristics.

### 2.2. Forms of Community Participation

According to the current research findings, as well as two special characteristics of “mobility” and “old age”, older migrants’ community participation can be categorized into three forms of welfare, development, and organizational participation.

Welfare participation refers to the use of public facilities and social services by older migrants [37], including community health services, senior privilege, and elderly care, which focuses on older migrants’ welfare rights and their access to community services. Health services are provided in accordance with the National Basic Public Health Service Specification (Third Edition) [38], which indicates older migrants can receive services, such as physical examinations, including pulse/blood pressure and vision/hearing tests, routine blood/urine and blood sugar tests, management of chronic illness, guidance of healthy lifestyle, and maintenance of health archives. In accordance with local practices, senior privilege is normally accessed with a privilege card, which entitles older migrants to local benefits and social services when they have submitted relevant documents to obtain eligibility [39]. Elderly care consists of various local services, including meals or canteens, bathing, housekeeping, holiday visits, haircuts, chatting, daily care, etc., which constitute utilization of community care services by older migrants.

Development participation refers to older migrants’ involvement in voluntary work and other public service activities, including political advice at the local community level [40]. It focuses on older migrants’ attention and participation in public affairs and community development. Voluntary work is altruistic, comprising community security patrols, dispute mediation, environmental sanitation/trash sorting, neighborhood assistance, disease prevention and control, education of younger generations (excluding their own grandchildren), etc. [41]. Political advice is mainly given to community committees or estate service enterprises on public affairs, such as design of public spaces, optimization of social services, and configuration of service facilities [42].

Organizational participation refers to older migrants’ participation in various types of social organizations at the community level, examining whether and to what extent older migrants have transferred their participation in the private family space to the public community space [43]. Specifically, organizational participation involves joining community associations, educational activities organized by community colleges and universities for the elderly, community security patrol groups, people’s mediation committees, public services, sports and recreation, and folklore/folk cultural organizations.

The three forms of community participation reflect two special features of old age and mobility, each with a different emphasis. Welfare participation underlines the increase in “self” interests, reflecting the implementation of local welfare policies and the realization of participation rights. Development participation prioritizes an increase in “others’” interests, highlighting the productive value of older migrants’ community participation in enhancing community unity. Organizational participation considers the protection of both “self” and “others’”, interests, focusing on the transformation from occasionally spontaneous to continuously organizational participation in community activities. These three types all mirror the social and public nature of community participation; their analysis can present a more comprehensive picture of the protection of participation rights, the reproducibility of social relations, and the formation of a sense of community among older migrants. Such analysis gives an indication of older migrants’ life conditions in a new environment.

## 3. Materials and Methods

### 3.1. Research Design

In this study, we adopted a quantitative research method to examine older migrants’ participation in urban communities and its key influences. We chose to focus on the 14 urban districts in 4 cities, including XS, BJ, YH, and LP districts in Hangzhou City, HS, JB, BL, and YZ districts in Ningbo City; OH and LW districts in Wenzhou City; and XZ and PZ districts in Jiaxing City. There were three reasons for the choice of the sample sites. First, the four cities are situated in the Yangtze River Delta region, which has experienced strong economic development and has a healthy living environment and a high level of social welfare. Second, the cities in this region had a larger number of migrants compared with other cities. According to the Zhejiang Province Statistical Yearbook 2020 [44], the migrant population in Hangzhou, Ningbo, Wenzhou, and Jiaxing accounted for 23.23%, 28.77%, 10.50%, and 24.23% of the total residents, respectively. Besides, the migrant populations in these sampled districts were higher than the city’s average annual inflow of migrants. For example, in 2019, the proportion of migrants in BJ (38.86%), XS (25.83%), and YH (38.56%) was 15.63%, 2.6%, and 15.33% higher, respectively, than the average of Hangzhou City. Third, the 14 selected urban areas had local administrative resources to help the researchers carry out the household survey, targeting migrant households with individuals aged 60 and above who had lived in the community for six months or more.

### 3.2. Data Collection

This questionnaire survey was designed to provide a comprehensive picture of the overall participation of older migrants in urban communities and the similarities and differences between the three forms of community participation. It comprised 45 questions, covering three sections of basic information on older migrants, typical patterns of community participation, and the evaluation of the living environment. Prior to the main survey, a pilot survey was conducted with 45 older migrants in one community of BJ district. As a result of the pilot survey, modifications were made to some questions in the survey in order to ensure that all questions were understood and accepted. The final questionnaire survey was carried out by face-to-face interviews between June and September 2020, and older migrant participants were selected based on migrant registrations. To compensate for missing respondents caused by delay in the update of the registration information, a snowball sampling method was employed to get the target population involved. A total of 1216 questionnaires were sent out, of which 1105 were valid after excluding incomplete questionnaire or incoherent responses. The overall response rate was 90.87%, as shown in Table 1.

### 3.3. Data Analysis

#### 3.3.1. Dependent Variables

The dependent variables refer to the three forms of community participation: welfare participation, development, and organizational participation. Regarding community health services, elderly care services, and senior privilege at the local level, receiving at least one of these benefits was assigned a value of “1” (“0” otherwise). Participation in any volunteering activities or providing community advice was assigned a value of “1” (“0” otherwise). Participation in social organizations, old age associations, or educational institutions in the community was assigned a value of “1” (“0” otherwise). Community participation in at least one of the three forms (welfare, development, or organization) was assigned a value of “1” (“0” otherwise).

#### 3.3.2. Independent Variables

The independent variables present a view of health status, human capital, and environmental characteristics. The three indicators were used to assess health status were self-assessed health, self-care capability, and chronic illness risk. Self-assessed health was assigned a value of 1–5 points, with 1 representing “very unhealthy” and 5 being “very healthy”. A value of “1” was assigned to the full capability of self-care, whereas “0” was assigned to impaired self-care capability. No chronic disease was given a value of “0”, or “1” otherwise.

Human capital was assessed by three indicators: educational attainment, vocational skills, and local language acknowledgement. Educational attainment consists of “primary school and below”, “middle and high school (vocational high school)”, and “tertiary institutions and above”. Vocational skills with relevant qualification and certificates were assigned a value of “1” (“0” otherwise). knowledge of the local language was assigned a value of “1” (“0” otherwise).

Environmental characteristics were evaluated by three indicators of urban inclusion, community support, and resident friendship. The specific questions were: “I think this city treats people from other cities equally”; “I think local community and organizations is friendly to migrants”; and “I think local residents are friendly to migrants.” These variables were dichotomous, with “Yes” being assigned a value of “1” and “0” for “No”.

#### 3.3.3. Control Variables

Based on the existing research findings on influences and the available data, eight control variables were selected to reflect the socioeconomic characteristics of the population, including gender, age, political identity, type of household registration, place of migration, migration period, living conditions, and self-assessed economic status. The last variable was assigned a value on a 5-point scale, with 1 representing “very poor” and 5 being “very wealthy”. “City-to-city migration” was assigned a value of “0”, whereas “interprovincial migration” was assigned a value of “1”. In terms of migration period, “<5 years” in the local community was assigned a value of “0”, and “≥5 years” was assigned a value of “1.” All other variables were dichotomous.

#### 3.3.4. Regression Analyses

Because the independent variables are all dichotomous, a binary logistic model was adopted for regression analysis. The empirical model was developed as follows:

$$\ln\left(\frac{p_{i}}{1-p_{i}}\right)=\alpha_{0}+\alpha_{1}X_{ij}+\beta Z_{ij}$$

where $p_{i}$ denotes the probability that the *i*th older migrant participates in local community; $X_{ij}$ denotes the independent variables of health status, human capital, and environmental characteristics; $Z_{ij}$ denotes other control variables; and *β* is the coefficient matrix. In this study, we examined the main factors shaping older migrants’ community participation in urban areas, specifically observing whether coefficient $a_{1}$ was positive or negative/large or small and whether associations were statistically significant. In this study, we aimed to investigate the overall community participation of older migrants, as well as the key influences on three forms of community participation. As a consequence, five binary regression analysis models were applied. M1 and M2 were regression analyses of the general community participation. M1 incorporated independent variables such as health status, social capital, and environmental characteristics, whereas M2 incorporated control variables based on M1. M3, M4, and M5 were regression analyses of the three forms of community participation: welfare, developmental, and organizational. The chi-square values of M1 to M5 were 155.34, 231.2, 190.13, 210.13, and 159.36, respectively, all of which reached a significance level of *p* < 0.001, indicating that the regression models were valid overall and that the independent variables are valuable to explain the influences of older migrants’ community participation.

## 4. Results

Table 2 shows survey results associated with independent and control variables that might affect older migrants’ community participation. In terms of the independent variables of health status, the mean value of older migrants’ self-assessed health was 3.8878 and equal to “relatively healthy”, with those who had full self-care capability accounting for 78.64%. Although older migrants with chronic illness accounted for 55.93%, these disabilities did not create a barrier to their autonomous mobility, and they were entitled to health selectivity [45]. Concerning the independent variables of human capital, 55.57% of older migrants had completed only elementary school or below, whereas only 8.69% held professional or technical vocational qualifications and 31.95% were familiar with the local language. This indicates that the capacity of older migrants’ human capital was rather limited. According to independent variables of environmental characteristics, the mean value of urban inclusion was 0.6118, which is higher than the mean value of community support (0.3792) and of resident friendship (0.3783), meaning that the dynamics of urban inclusion were better in comparison with those of community support and resident friendship. With respect to the control variables, 51.58% of older migrants were female, and 75.88% were younger than 70 years old, which is in line with the characteristics of age-selective mobility [46]. Those with agricultural household registration accounted for 69.59%, whereas 87.51% lived with their rural-to-urban families. Interprovincial migrants accounted for 79.91%, and 62.44% had lived in the community for less than 5 years. Only 8.9% of older migrants were members of the Communist Party of China; their economic status was self-assessed as average, with a mean value of 3.1548.

Table 3 presents the descriptive statistics of older migrants’ community participation. The rate of community participation is the ratio of older people who participate in community activities relative to total older migrants. Only 364 (32.94%) of older migrants had participated in the local community, representing a relatively low level of community involvement. There were certain differences in terms of migrants’ affection for the three forms of community participation, with the highest rate of 24.71% participating in welfare activities, followed by 15.2% in development activities and 8.96% in organizational activities. In particular, participation in community health services, privilege card application, and elderly care services reached more than 12.00%; participation in voluntary services and political advice reached 9% or over; and participation in social organizations varied considerably, with 8.40% joining community social organizations, in contrast to less than 2% participating in educational organizations and old age associations.

Table 4 displays five binary regression models (M1 to M5) that underline correlations between the independent and dependent variables. Specifically, M1 inputs all variables of health status, human capital, and environmental characteristics to measure their impacts on general community participation. Among three indicators of health status, self-care capability and chronic illness risk had a significant effect on community participation. The proportion of older migrants with full self-care capability was 1.420 times higher than that with impaired self-care capability in community participation. Those with chronic illness were 1.747 times more likely to get involved in the local community compared with others. With regard to human capital, educational attainment and local language knowledge significantly affected the overall level of community participation. There was a negative correlation between primary school education or below and community participation. In other words, the higher the educational attainment, the higher the community participation. Older migrants who understood the local language had higher levels (1.402 times) of community participation than those who did not. The three indicators of environmental characteristics, urban inclusion, and resident friendship had positive effects on community participation, which means that older migrants in more inclusive and friendly communities exhibited higher levels of community participation.

M2 includes control variables to estimate correlations between all variables in M1 and community participation. This model indicates that there were no substantial changes in associations between independent variables and dependent variables, except for significant effects of the same variables. The control variables of gender, household registration, political identity, living conditions, migration place, and migration period had significant effects on older migrants’ community participation. Male participation was only 67.2% of female participation, and migrants in households registered as non-agricultural were 1.777 times more likely to participate in community activities compared to those with agricultural household registration. Community participation was 2.715 times higher among members of the Communist Party of China than among non-members. Older migrants living alone were found to have higher community participation than those living with their family (spouse or children). City-to-city migration within the same province had direct influences on levels of community participation, and those who migrated to the local community more than 5 years ago had a greater likelihood of participating in community activities. In other words, levels of community participation were positively correlated with the location and the period of migration.

M3 shows associations between independent variables and the form of welfare participation. This model indicates that self-care capability, chronic illness, urban inclusion, and resident friendship were significant predictors of welfare participation. Older migrants with full self-care capability and chronic illness were more likely to participate in community welfare issues. Meanwhile, better urban inclusion and resident friendship were found to have a positive effect on welfare participation. The value of urban development and the design of public policy normally reflect a city’s transparency and acceptance, which determines the accessibility of community public services for older migrants. Communication with neighbors often facilitates exchange and transmission of public service information that can guide older migrants to utilize such public service resources. However, all variables of human capital were measured to have no correlations with welfare participation, suggesting that older migrants’ education and qualification have little effect on their receipt of welfare benefits. The control variables of gender, household registration, political identity, migration space, and migration period were significantly linked to older migrants’ welfare participation. The proportion of male participation was 61.6% of that of female participation, and that of older migrants with non-agricultural household registration was 1.676 times higher than that of migrants registered in agricultural households. Members of the Communist Party of China had a greater likelihood of participating in local welfare programs than non-members. Similarly, city-to-city migrants and those living in the local community for more than 5 years were more likely to receive community welfare assistance.

M4 measures the impacts of independent variables and control variables on the form of development participation. In terms of independent variables, this model indicates that education attainment, local language knowledge, and environmental characteristics had a significant effect on older migrants’ developmental participation. The development participation rate of older migrants who completed primary school or below was only 28.3% of that of older migrants with a college education or higher. A positive correlation was found between knowledge of the local language and participation in community development. All factors of environmental characteristics, including urban inclusion, community support, and resident friendship, had direct effects on development participation. In other words, those living in an inclusive or friendly community and maintaining good neighborhood relationships reported higher levels of involvement in local development projects. The status of physical and mental health was found to have no correlation with development participation. With regard to the control variables, household registration, migration place, and migrant period were significantly linked to development participation. Those who had non-agricultural household registration, had migrated within the same province, moved to the local community more than 5 years ago, and living with their family tended to have greater participation in community development.

M5 examines associations of independent variables and control variables with dependent variables. Among independent variables, this model showed that educational attainment, urban inclusion, and community support were significant predictors of organizational participation. There was a negative correlation between primary education and organizational participation. An inclusive city or a friendly community had a positive effect on older migrants’ engagement in organizational events. This indicates that there might be limited information on voluntary and community organizations among older migrants. Besides, the governance and threshold for participating in some types of organizations probably restricted the elderly’s involvement and participation. However, the three indicators of health status were found to have no significant impacts on organizational participation. In terms of control variables, household registration, migration space, and migration period had significant influences on organizational participation. The participation rate in community organizations of those with non-agricultural household registration was 6.405 times higher than that of other older migrants. City-to-city migrants within the same province were more likely to get involved in such local organizations. Meanwhile, those who moved into the local community more than 5 years ago had greater engagement in organizational activities. The remaining indicators, including gender, age, political identity, living conditions, and self-assessed economic status, were measured to have no correlations with organizational participation.

## 5. Discussion

The real state of community participation among older migrants tends to be “paradigms lost”, which means that they live in the physical space of a community without participating in the social life. Most surveyed older migrants only regarded their community as a place of residence rather than one for relationship construction, which is basically consistent with other relevant studies on older adults or migrants [25,47]. Older migrants might be unable to receive required services from both family members and local government because residential segregation excludes them from public services and welfare privileges. This is undoubtedly a form of depriving older migrants of their rights, introducing a barrier to the development of an age-friendly society. Often, community organizations serve as the stage for native resident performance rather than as a platform for older migrants’ collaborative participation. Older migrants lack subject consciousness in urban communities, which they treat as temporary dwellings rather than permanent homes. Likewise, for local community organizations, older migrants are considered “outsiders”.

Given the levels of community participation, female older migrants were more dedicated than male older migrants, and “urban-to-urban” migrants had a greater likelihood of participation than “rural-to-urban” migrants. These results are consistent with previous findings [25,27]. Limited participation among “rural–urban” older migrants is due to the deficiency in the awareness of participation rights and the capability to participate in community activities among rural older people in China. The level of community participation was higher for those with political identity associated with the Communist Party of China, as party membership implied a higher capacity to integrate resources and a stronger sense of responsibility for community development. Those who lived with family members (their spouse or children) were less likely to participate in the community, possibly because caring for grandchildren took up most of their time and energy rather than community participation. Family support might create an alternative to the pursuit of community participation. This suggests that an increase in community participation among older migrants requires the joint effort of family support to release older people for community activities as much as possible. Interprovincial migration brings intense changes in life circumstances and considerable shocks in cultural practices to older people. Furthermore, the provision of public services based on household registration and the “dragnet effect” on social relations [48] limited community participation among province-to-province older migrants. In addition, it takes time for older migrants to adapt to their new environment [12,49], and the accumulation of residence time facilitates the development of personal interactions and the exchange of participation chances within local communities. Therefore, it is important to establish mechanisms for the delivery of elderly care services and welfare in accordance with the number of older residents. It is also necessary to promote the participation of “rural-to-urban” older migrants, especially when they migrate from another province. It is indispensable to develop community-based care services for older people, as the community offers more opportunities for the provision of elderly care welfare, community organizational resources, local language communication, and traditional culture and customs.

The higher likelihood of community participation among those with full self-care capability or with chronic illness suggests that older people engage in community participation regularly when they have a certain level of mobility. Specifically, the fact that older people with chronic illness were more likely to participate in community activities implies that they have stronger health consciousness and were more proactive accessing community public services. Therefore, it is necessary to ensure flexible community health services and proactive delivery of welfare and elderly care services can benefit older people, especially those with impaired mobility. Higher educational attainment and knowledge of the local language were significant to community participation, which is consistent with prior research [50]. Both the formation of community participation awareness and the enhancement of competencies require a certain level of literacy. It is easier to learn the local language than improve educational attainment at older ages, as local languages could be acquired during adaptation to the local community. Additionally, community participation connects residents and community organizations through language, and familiarity with the local language facilitates this connection [3,4,9]. Therefore, communities with a high proportion of older migrants can provide services for migrants to learn the local languages. Positive evaluation of urban inclusion and resident friendship significantly increased community participation, which is in line with relevant studies [51,52]. Urban inclusion essentially represents the value of urban development. The process of formulating and implementing public policies without household registration could reflect care for older migrants. Resident friendship indicates the acceptance of older migrants by regional residents in terms of attitudinal perceptions, daily interactions, and public activities. Indeed, mutual promotion between environmental inclusion and community participation offers more opportunities for community participation, whereas community participation enhances the perception of environmental inclusion.

The independent variable of urban inclusion and the control variables of household registration and migration place had significant effects on the three types of participation. The effect of urban inclusion showed that external support in cities for older migrants to participate in the community surpassed the household registration-based mechanisms of elderly services and welfare provision. The effects of the latter two variables demonstrate that public service provision based on household registration limited older migrants’ community participation, whereas the accumulation of social relations improved community participation. None of the independent variables characterizing human capital had a significant effect on welfare participation, suggesting that welfare participation was mainly constrained by environmental inclusion and the health status of older migrants. The research results are of great significance for policy making. First, it is necessary to provide preferential treatment and services to older adults in communities and explore ways to transfer welfare for older migrants between cities so that elderly care services, and welfare and policies are be delivered to all older adults in the community. Secondly, it is important to eliminate the mechanism by which older migrants are provided services and welfare based on household registration [53]. A welfare and service provision system linked to years of residence would offer older migrants treatment equal to that of other local older adults [54]. Thirdly, the acceptance of “outsiders” by regional registrants in the community should be increased. The fact that all the independent variables characterizing health status had no significant effect on development participation and organizational participation suggests that community participation requires not only being healthy but also awareness of participation and more opportunities for older migrants. Therefore, community organizations and residents should be more accepting of “outsiders”. The threshold for community participation based on household registration should be canceled to improve the accessibility of participation and governance for older migrants.

## 6. Contributions and Limitations

In this study, we paid special attention to older migrants’ participation in community actions, which provided an illuminating insight into older migrants’ life in a new environment. The discourse on community participation frames the life experiences of older migrants and the ways they integrate with unfamiliar surroundings. This is significant in the context of national migration and suggests the need for further research on the role of participation in healthy ageing in local communities. In this study, we classified older migrants’ activities and interactions into three types participation—welfare, development, and organizational participation—which refined the understanding of life transitions, particularly in the context of welfare protection, social ties, and collective consciousness. The identification of different influences on each form of participation will contribute to an improvement of older migrants’ attentiveness to individual or organizational activities. In addition, the research findings highlight the importance of health, capability, and opportunity to active participation in different forms of activities, proposing new directions and emphasizing their roles in migration and resettlement in later life.

Although this study contributes to the understanding of older migrants’ participation in different types of activities, there are some limitations. First, this research investigated older migrants across Zhejiang Province, where local communities have a comparative advantage to provide a broader range of social services or public activities. It is possible for certain factors to cause bias in responses to a survey. Therefore, caution should be exercised when generalizing the research findings. Secondly, although certain considerations were given to previous research on local people’s participation, this study was centered on the participation of the older migrant population in community activities. Further research is needed to specifying differences in community participation between local older people and older migrants so that actions can be easily adopted and best practices can be introduced. Finally, because most survey respondents remained capable of participating, whereas those in need of complete assistance were unable to engage in development and organizational activities (but could participate in some welfare services), the variable of self-care capability was defined with two codes—impaired self-care capability and full self-care capability—which might impact the research results.

## 7. Conclusions

Active aging recognizes older adults’ rights to obtain equal opportunities to engage in all aspects of social life as they age. Under the division of public service provision dependent on the status of household registration, older migrants may be excluded from the social protection mechanisms present in their place of domicile because of “migration”. If the local social environment is not inclusive enough to enable all residents to share the fruits of development, older migrants will be caught in an awkward situation of not being able to participate in either their original community or their relocated community. This results in a tendency of older migrants to becoming “paradigms lost” in community participation, especially “rural-to-urban” older migrants who migrated from one province to another. It should be recognized congruously across the whole society that older migrants have the need and right to participate in the local community.

Health is the foundation of community participation. In practice, maximization of older adults’ autonomy should be a main objective of local communities, ensuring that they are covered by community health services in order to reinforce the health foundation of their community participation. In terms of human capital, the ability to participate is a prerequisite for older migrants’ community participation, and the provision of services for local language learning is an effective way to improve the accumulation of human capital among this group. Opportunities are needed for older migrants’ community participation. Only when there are opportunities for community participation can older migrants put their thoughts and abilities of participation into action. This require an effective implementation of the existing policies on senior privilege and elderly care services. It also requires new policies to allow for the transfer of welfare benefits and entitlements between cities and to build systems connecting the provision of public services with length of residence in a community for new migrants. In addition, the transparency of urban cities and the friendly behavior of local residents can promote community solidarity, which strengthens the external environment to support older migrants’ participation.

## Figures and Tables

**Table 1:** Different levels of response rates across four cities.

City	Survey Samples		Valid Samples	
	Number	Percentage (%)	Number	Percentage (%)
Hangzhou	376	30.92%	363	32.85%
Ningbo	347	28.54%	327	29.59%
Wenzhou	228	18.75%	193	17.47%
Jiaxing	265	21.79%	222	20.09%
Total	1216	100.00%	1105	100.00%

**Table 2:** Descriptive statistics of independent and control variables.

	Variable	Code	Percentage (%)	Mean	SD	Min	Max
Independent variables	**Health status**						
	Self-assessed health	1 = Very unhealthy	0.90%	3.8878	0.8608	1	5
		2 = Relatively unhealthy	5.25%				
		3 = Average	22.08%				
		4 = Relatively healthy	47.69%				
		5 = Very healthy	24.07%				
	Chronic illness	0 = No	44.07%	0.5593	0.4967	0	1
		1 = Yes	55.93%				
	Self-care capability	0 = Impaired self-care capability	21.36%	0.7864	0.4100	0	1
		1= Full capability of self-care	78.64%				
	**Human capital**						
	Educational attainment	1 = Primary school and below	55.57%	1.4960	0.5945	1	3
		2 = Middle and high school (vocational high school)	39.28%				
		3 = Tertiary institutions and above	5.16%				
	Vocational skills with relevant qualification and certificates	0 = No	91.31%	0.0869	0.2818	0	1
		1 = Yes	8.69%				
	Knowledge of local language	0 = No	68.05%	0.3195	0.4665	0	1
		1 = Yes	31.95%				
	**Environmental Characteristics**						
	Urban inclusion	0 = No	38.82%	0.6118	0.4876	0	1
		1 = Yes	61.18%				
	Community support	0 = No	62.08%	0.3792	0.4854	0	1
		1 = Yes	37.92%				
	Resident friendship	0 = No	62.17%	0.3783	0.4852	0	1
		1 = Yes	37.83%				
Control variables	Gender	0 = Female	51.58%	0.4842	0.5000	0	1
		1 = Male	48.42%				
	Age	0 = 60–69	78.55%	0.2145	0.4107	0	1
		1 = ≥70	21.45%				
	Type of household registration	0 = Agricultural	69.59%	0.3041	0.4602	0	1
		1 = Non-agricultural	30.41%				
	Living with family members	0 = No	12.49%	0.8751	0.3307	0	1
		1 = Yes	87.51%				
	Migration place	0 = City-to-city migration	20.09%	0.7791	0.4009	0	1
		1 = Interprovincial migration	79.91%				
	Migration period	0 = <5 years	62.44%	0.3756	0.4845	0	1
		1 = ≥5 years	37.46%				
	Political identity	0 = Public	91.1%	0.0887	0.2844	0	1
		1 = Member of Communist Party of China	8.9%				
	Self-assessed economic status	1 = Very poor	0.36%	3.1548	0.6143	1	5
		2 = Relatively poor	9.68%				
		3 = Average	65.61%				
		4 = Relatively wealthy	22.81				
		5 = Very wealthy	1.54%				

**Table 3:** Number and percentage of participants in the three forms of participation.

	Number of Participants (*n*)	Percentage of Participation (%)
**Welfare participation**	273	24.71%
Health services	161	14.60%
Privilege card application	137	12.40%
Elderly care services	94	15.70%
**Development participation**	168	15.20%
Voluntary services	98	8.9%
Political advice	116	10.5%
**Organizational participation**	99	8.96%
Community social organizations	93	8.4%
Old age associations	21	1.9%
Education organizations	18	1.6%
**Total**	364	32.94%

**Table 4:** Model analysis of factors influencing community participation among older migrants.

Variable	M1 (General Participation)		M2 (General Participation)		M3 (Welfare Participation)		M4 (Development Participation)		M5 (Organizational Participation)	
	B	exp (B)	B	exp (B)	B	exp (B)	B	exp (B)	B	exp (B)
**Health status**										
Self-assessed health	0.012	1.012	0.022	1.022	−0.016	0.984	0.098	1.103	0.158	1.171
Full self-care capability	0.351	1.420 *	0.400	1.491 *	0.821	2.273 ***	0.324	1.732	0.278	1.320
Chronic illness (None)	0.558	1.747 **	0.502	1.652 **	0.500	1.649 **	0.227	1.254	0.421	1.523
**Human capital**										
Educational attainment (Tertiary institutions and above)										
Primary school and below	−1.253	0.286 ***	−0.668	0.512 *	−0.689	0.502	−1.261	0.283 **	−1.054	0.349 *
Middle and high school	−0.436	0.647	−0.044	0.957	−0.210	0.810	−0.224	0.799	−0.397	0.673
Attained qualification certificates	0.247	1.281	0.102	1.108	0.172	1.187	0.476	1.621	0.199	1.820
Knowledge of local language	0.338	1.402 *	0.262	1.300*	−0.054	0.948	0.590	1.804 **	0.108	1.898
**Environmental characteristics**										
Urban inclusion	0.604	1.829 ***	0.575	1.778 **	0.586	1.797 **	0.597	1.817 *	1.166	3.209 **
Community support	0.267	1.306	0.253	1.228	0.318	1.374	0.410	1.508 *	0.413	1.512 *
Resident friendship	0.432	1.540 **	0.406	1.500*	0.506	1.659 **	0.467	1.595 *	0.279	1.321
**Control variables**										
Gender (female)			−0.397	0.672 **	−0.414	0.616 *	−0.054	0.947	−0.200	0.819
Age (<70)			0.079	1.083	0.311	1.365	0.067	1.069	−0.299	0.742
Type of household registration (agricultural)			0.575	1.777 **	0.517	1.676 *	0.961	2.614 ***	1.857	6.405 ***
Interprovincial migration (city-to city migration)			−0.729	0.482 ***	−0.701	0.496 ***	−0.664	0.515 **	−0.658	0.518 *
Migration period ≥5 years			0.773	2.166 ***	0.788	2.199 ***	0.812	2.253 ***	0.820	2.271 **
Political identity (non-members of Communist Party of China)			0.999	2.715 ***	0.831	2.296 **	0.442	1.526	0.453	1.572
Living with family members ( no)			−0.509	0.601 *	−0.383	0.682	−0.639	0.528 *	0.274	1.315
Self-assessed economic status			−0.018	0.982	−0.197	0.821	0.106	1.112	−0.302	0.704
Constant	−1.357	0.257	−1.144	0.319	−1.293	0.275	−2.447	0.087	−3.883	0.021
Sample size	1105		1105		1105		1105		1105	
Nagelkerke R²	0.183		0.263		0.235		0.302		0.296	

Reference groups in parentheses; *, **, and *** indicate significance at the 5%, 1%, and 0.1% levels, respectively.

## Data Availability

The data presented in this study are available from the corresponding author on reasonable request. The data are not publicly available due to their containing information that could compromise the privacy of research participants.
